# Influence of Spatial Inhomogeneity of Detector Temporal Responses on the Spectral Fidelity in Continuous Wave Cavity Ringdown Spectroscopy

**DOI:** 10.3390/s19235232

**Published:** 2019-11-28

**Authors:** Zhensong Cao, Zhixin Li, Fei Xu, Yongqian Wu, Zixin Zhou, Zhaomin Tong, Weiguang Ma, Wenyue Zhu

**Affiliations:** 1 Key Laboratory of Atmospheric Optics, Anhui Institute of Optics and Fine Mechanics, Chinese Academy of Sciences, Hefei 230031, China; zscao@aiofm.ac.cn (Z.C.); zhuwenyue@aiofm.ac.cn (W.Z.); 2 School of Software, Shanxi University, Taiyuan 030006, China; 3 State Key Laboratory of Quantum Optics and Quantum Optics Devices, Institute of Laser Spectroscopy, Shanxi University, Taiyuan 030006, China; 4 Collaborative Innovation Center of Extreme Optics, Shanxi University, Taiyuan 030006, China; 5 Institute of Optics and Electronics, Chinese Academy of Sciences, Chengdu 610209, China; wyq95111@sina.com

**Keywords:** cavity ringdown spectroscopy, spatial effect, temporal response, spectral fidelity

## Abstract

Due to their advantages of having a wide bandwidth, low cost, and being easy to obtain, traditional photodetectors (PDs) are being widely applied in measurements of transient signals. The spatial inhomogeneity of such PD temporal responses was measured directly to account for the PD spatial effect of decay rate due to poor alignment in continuous wave cavity ringdown spectroscopy (CW-CRDS) experiments. Based on the measurements of three PDs (i.e., model 1611 (Newport), model 1811 (Newport), and model PDA10CF-EC (Thorlabs)), all the temporal responses followed a tendency of declining first and then rising, and steady platforms existed for the last two PDs. Moreover, as we expected, the closer the PD center was, the faster the response. On the other hand, the initial shut-off amplitude generally reached a larger value for a faster temporal response. As a result, the spatial effect can strongly influence the spectral line shape and value, which will introduce more errors into the precise measurements of spectral parameters using the CRDS technique if this effect is not considered. The defined effective detection area (EDA) of the PDs, which was close to the active area given by manufacturers, was the key parameter that should be paid more attention by researchers. Therefore, the PD should be aligned perfectly to make sure that the EDA covers the laser spot completely.

## 1. Introduction

Molecular spectroscopy techniques, which have advantages in terms of sensitivity, resolution, and linearity, are crucial in field applications and fundamental physical measurements. For example, the line strengths of target transitions are required for ultra-trace gas detection in the semiconductor fabrication process [1] and the isotope ratio measurement of gaseous molecules in geological surveys [2], and their accuracies must be as precise as possible. The Boltzmann constant can be determined by accurately measuring the Doppler broadening width of an absorption line based on the Doppler broadening thermometry in the field of precise measurement [3]. Accurate values for pressure broadening and frequency shift coefficients of the target transitions of molecular gas are necessary to get a precise measurement of gas concentration in the lower stratosphere and upper troposphere based on a light detection and ranging (LIDAR) system [4]. Therefore, the measurement of an absorption line, with high fidelity in terms of frequency resolution, spectral line shape, and absorption coefficient, is necessary.

Cavity ringdown spectroscopy (CRDS) is a sensitive and quantitative absorption spectroscopy method that is based on the decay rate measurement of a laser beam trapped in a high-finesse optical cavity. Since the first demonstration by O’Keefe and Deacon in 1988 [5], CRDS has been used for highly sensitive absorption measurements of weak transitions or rarefied species in various environments and configurations [6,7,8,9,10,11,12,13,14,15,16,17,18,19,20,21,22,23]. Benefiting from the high-finesse optical cavity, the interaction length between the light and target substances can be enhanced efficiently [24,25]. Compared with other sensitive absorption techniques, CRDS has an additional advantage that the absorption is measured on an absolute scale [26].

Initially, a wavelength tunable, pulsed laser is used for the determination of the cavity medium’s absorption spectrum. Generally, the laser bandwidth exceeds the cavity mode spacing, thereby giving rise to spectral overlap between the probe laser and several cavity resonances. Such a coupling between the broadband laser and the cavity causes a multimode excitation and results in a multi-exponential decay, which means the signal-to-noise ratio (SNR) and frequency resolution of the spectral signal are quite low. The conditions under which CRDS can be used for quantitative diagnostics of molecular species were examined and determined by Zalicki and Zare [27]. Hodges et al. found that failure to account properly for the laser bandwidth would lead to systematic errors in the number of densities determined from measured ring-down signals [28]. Therefore, a high-fidelity absorption spectrum cannot be realized by use of a wideband, laser-excited CRDS.

In 1996, Lehmann proposed a CRDS setup using a narrow linewidth continuous wave (CW) laser instead of pulsed lasers, which can provide single mode excitation and high SNR [29]. In order to increase the spectral fidelity of continuous wave cavity ringdown spectroscopy (CW-CRDS), a series of works were performed by Hodges’ group [30,31,32,33]. Firstly, a refined CRDS, called frequency-stabilized CRDS (FS-CRDS), was proposed by stabilizing the cavity length to a laser reference, which can produce a linear, stable, and accurate frequency axis underneath the absorption spectra. Benefiting from the superior features of the FS-CRDS, the line strength and pressure broadening and shift coefficients of the five transitions were measured with a frequency resolution of 1 MHz [30].

In order to address weaker transitions in CRDS experiments, a pair of high-reflectivity cavity mirrors are required. However, the scattering due to cavity mirror surface defects will cause a wavelength-dependent loss in the order of ppm, and more importantly, it can produce an optical frequency-dependent finesse drop, which originates from the transverse mode coupling [34,35,36,37] and spatial inhomogeneity of mirror reflectivity due to coating [38]. In 2015, a pin hole was inserted into the cavity and the transverse mode coupling was reduced effectively in a noise-immune, cavity-enhanced, optical heterodyne molecular spectroscopy (NICE-OHMS) experiment [39].

As highlighted in the previous descriptions, although efforts had been undertaken to obtain high-fidelity and high-sensitivity spectra measurements, the temporal response of a photodetector (PD) is another key parameter in the decay time measurement of CRDS that had not been considered. Until now, multiple types of detectors [40], such as photon multiplier tubes (PMT) and avalanche PDs, have been provided commercially and used to detect weak light with sub-ns response times, which makes them perfectly matched for use in CRDS. Besides these types of photodetectors, traditional PDs are also good options for temporal measurements due to their advantages of have a wide response bandwidth, being low cost, and readily available. For such a PD with a given bandwidth, the rising time from 10% to 90% of the amplitude can be calculated as *t_r_* = 0.35/BW according to the rule of thumb, where BW is the bandwidth.

As we know, the response time of a PD is limited by the capacitance of a p–n junction. In general, the larger the junction area means the larger the capacitance. A diameter smaller than 0.5 mm is required for a bandwidth in the order of 100 MHz. If such a detector is used in the related measurements, the spatial effect of the PD temporal response should be evaluated systemically and a result should be given for the selection and alignment of a special PD.

In this paper, the spatial effects of three PDs with different temporal responses are measured and evaluated, and then the PD spatial effects of the absorption spectrum in CW-CRDS experiments are measured. According to the measured results, we know that the highest detection efficiency and response bandwidth of PD can be guaranteed only in the effective detection area (EDA), which is slightly smaller than the area given by the datasheet. Outside of this range, the response characteristics decline, as moving away from the center position of the PD. On the other hand, neglecting the PD spatial effect leads to underestimations of intracavity loss in the CW-CRDS experiment. Therefore, for reliable and stable trace gas detection with CW-CRDS, one should make sure that the laser beam is aimed at the EDA of the PD and that the spot size is focused properly to match the size of this area.

## 2. Two-Dimensional Response Characteristics of Three Different PDs

In order to clearly understand the spatial effects of PD temporal responses, a series of measurements for three different detectors (i.e., models 1611 and 1811 from Newport Corporation and model PDA10CF-EC from Thorlabs) were performed. The parameters given by the manufacturers for the three PDs are shown in Table 1. Note that the DC output is evaluated for model 1611, the bandwidth of which is mainly limited by electronics.

The experimental system was based on a commercial erbium-doped fiber laser (Koheras Adjustik E15 PztS PM, NKT Photonics, Birkerød, Denmark) with a free running linewidth of 1 kHz (over 120 μs integration time) and a wavelength tuning range from 1530.8 nm to 1531.6 nm through temperature control. Firstly, we guided this laser emission passing through the fiber pigtailed acousto-optic modulator (AOM) (MT110-IIR20-Fio- PM0.5-J1-A, AA Opto Electronic, Orsay, France) with an announced rising–falling response time of 20 ns and its first-order diffraction emitted. The AOM was driven by a power-amplified voltage controlled oscillator (VCO) (MODA110-B51k-34, AA Opto-Electronic, Orsay, France), which was controlled by an integrated digital transistor transistor logic (TTL) radio frequency (RF) source to switch the laser beam on or off. In these measurements, the VCO was controlled by a 200 Hz square wave with an amplitude of 2 V. A spatially variable neutral density filter was used to control the laser power to desaturate the PD. Continuously, the laser beam was focused to a spot with a diameter of about 57 μm by a convex lens (*f* = 30 mm), which was measured by using a beam profiler (BP109-IR, Thorlabs, Newton, NJ, United States). In order to avoid the feedback interference, the optical devices were all tilted slightly away from the normal incidence. The PD mounted on a high-precision, three-dimensional translation stage was located at the focal point and the laser power was controlled to be about 0.4 mW. After each fixation of the position in the horizontal direction, the PD was moved from up to down with a fixed step. The data of transients were collected by a 200 MHz oscilloscope (TDS2024C, Tektronix, Shanghai, China) with a 2 GHz sampling rate.

**Figure 1:**
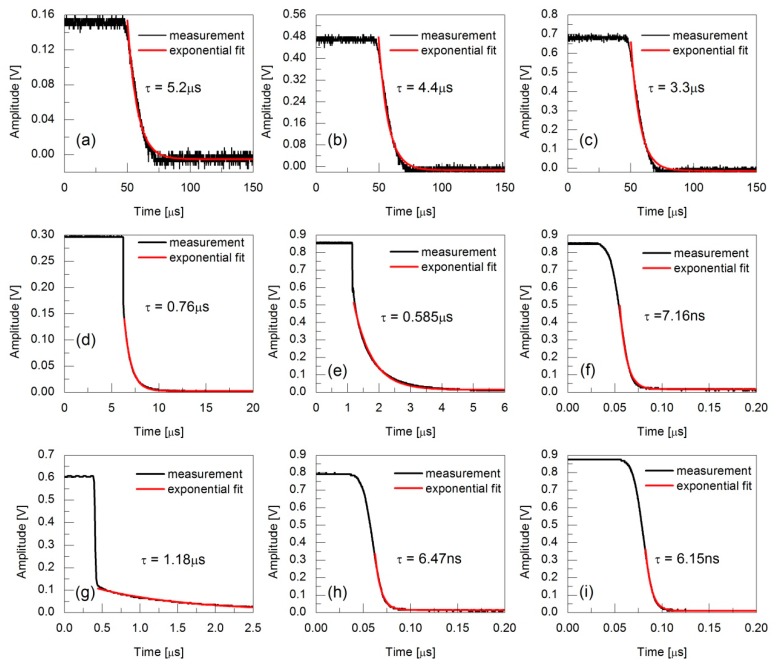
The transients in three typical positions of three detectors: (**a**–**c**) model 1611, (**d**–**f**) model 1811, (**g**–**i**) model PDA10CF-EC. (**a**–**i**) The data correspond to the PD positions 1 to 9 in Figure 2.

**Figure 2:**
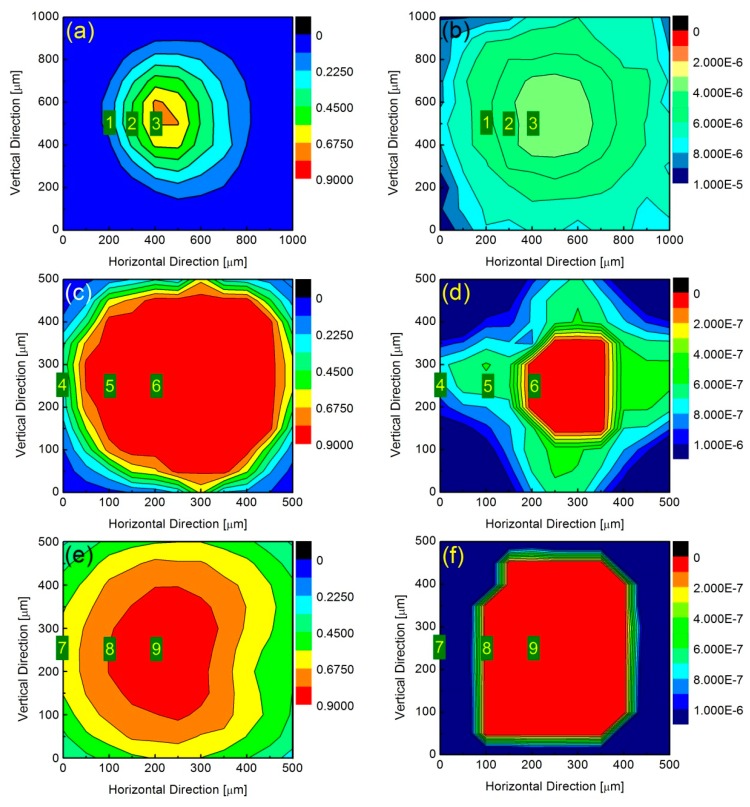
The two-dimensional contour plot of initial shut-off amplitudes and temporal responses as functions of vertical and horizontal positions of three different PDs. Panels (**a**), (**c**) and (**e**) show the initial shut-off amplitudes for the detectors of models 1611 (Newport), 1811 (Newport), and PDA10CF-EC (Thorlabs), respectively, and Panels (**b**), (**d**) and (**f**) show their corresponding ringdown times. The numbers 1 to 9 represent the measurement positions of Figure 1a–i.

A series of transients of beam interruption were collected when the PD was located at different positions, or in other words, when the light spot impinged on different parts of the PD. The measured transients in three typical positions of three detectors (i.e., Figure 1a–c for the DC output of model 1611, Figure 1d–f for model 1811, and Figure 1g–i for model PDA10CF-EC) are shown in Figure 1. Among the figures, the data from Figure 1a–i correspond to PD positions 1 to 9 in Figure 2, and the sampling step is 100 μm for each detector. The red curves show the corresponding single exponential fitting and the fitted decay times are shown as τ. The data curves in Figure 1a–c are thicker because there are no averages. The transients in these panels give the decay times in the order of μs due to the slow response of the DC output of model 1611. For Figure 1d,e,g, the transients are composed of an initial quick interruption and then a slow varied tail. The starting amplitude of the tail increases as the PD moves away. It is worth emphasizing that the initial shut-off amplitude increases to its maximum, but a relatively long decay tail still exists in Figure 1d,e for model 1811. However, the initial shut-off amplitude barely increases, but the decay time decreases to 7.16 ns in Figure 1e,f, where the initial shut-off amplitude is defined as the amplitude of the detector signal at the shut-off time. Such behavior confirms the fact that part of the laser beam hits the PD active area, and the other part hits the outer range in Figure 1d,e and all the light incidents on the active area in Figure 1f. The former contributes the voltage amplitude and quick interruption to the signal due to the higher photoelectric transformation efficiency and fast response, and the latter contributes the slow varied tail due to its opposite performance. For the other two detectors, as the decay time shortens, the initial shut-off amplitude increases. Generally, the experimental measurement of the transient is a convolution over the response time of the PD and the time delay of the cutoff process. The decay of the tail qualitatively represents the time response characteristic of PD, since the shut-off time of AOM is fairly quick at around 20 ns, as given by the datasheet. Although the measured decay times of transient responses in Figure 1f,h,i are 7.16 ns, 6.47 ns, and 6.15 ns, respectively, the shut-off times taken from 90% to 10% of the detector amplitudes are all close to 20 ns.

Figure 2 displays the two-dimensional contour plot of initial shut-off amplitudes (left column) and temporal responses (right column) as functions of vertical and horizontal positions of three different PDs, namely model 1611 (Newport), model 1811 (Newport), and model PDA10CF-EC (Thorlabs) from the upper to the lower rows, respectively. The units for color scales on the right side of the columns are voltage and seconds, respectively. In these panels, the data were acquired when the detectors were located at a serials of horizontal (x) and vertical (y) coordinates with a fixed step. The steps for the last two detectors were 50 μm and for the first detector it was 100 μm.

The settings for color scales and axis ranges for model 1611 are different from those of models 1811 and PDA10CF-EC, while the latter two are manually set to the same settings. For model 1611 (Figure 2a,b), both responses are lower compared to the other two PDs, since its DC bandwidth output is only 20 kHz. The closer the detector center is, the faster the response will be. Moreover, the shortest temporal response is larger than 3 μs, which indicates that this detector could cause large error when measuring time decay signals with time constants of several μs. The calculated rising time is 17.5 μs according to the previous empirical formula, which is around six times longer than the shortest measured value. Although the PD diameter is around 100 μm, there is still signal amplitude when the beam is 500 μm away from the center due to the ball lens having a diameter of 1.5 mm. However, for models 1811 and PDA10CF-EC, the calculated rising times are 2.8 ns and 2.3 ns, which are close to, but smaller than, the shortest measured temporal responses of 7.16 ns and 6.15 ns in Figure 2d,f, respectively. The reason for the larger measured values is due to the relatively slow response of AOM. As can be seen from Figure 2c,d, the measured initial shut-off amplitude shows a platform and a relatively steep edge for model 1811, however the contour lines for the temporal responses show square shapes in the detector’s outer range and circular shapes around the center. The response times are faster than 1 μs in the square area and are even faster than 0.1 μs in the circular area, with a diameter of 200 μm. For model PDA10CF-EC (Figure 2e,f), the initial shut-off amplitude increases slower than that of model 1811 when the laser spot is moved from the outside to the center of the PD. However, the response times show a sharp decrease within the circumference at about 150 μm from the center. Inside this circumference, the response times are faster than 0.1 μs. If we define the zones with response times smaller than 0.1 μs as the effective detectable areas (EDAs) of models 1811 and PDA10CF-EC, the EDAs are slightly smaller than the detectors’ officially stated active areas, which is partially explained by the remarkable size of the laser spots in both detectors.

## 3. PD Spatial Effect in the CW-CRDS Experiment

### 3.1. Experimental Setup

**Figure 3:**
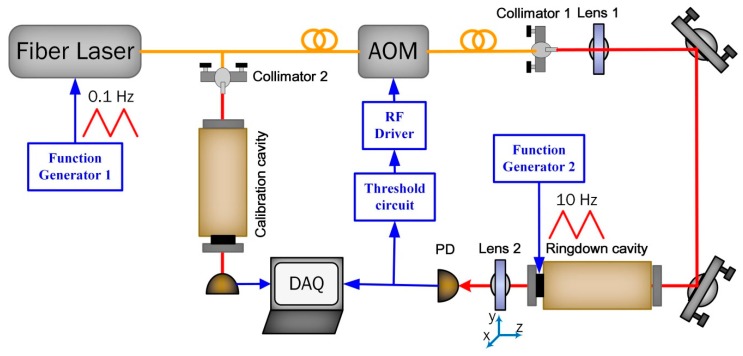
Setup designed for continuous wave cavity ringdown spectroscopy (CW-CRDS) experiments. Note: AOM = acoustic-optic modulator; DAQ = data acquisition card; PD = photodetector.

The experimental design of our CW-CRDS setup for the PD spatial effect measurement is schematically depicted in Figure 3. The system was still based on the previously mentioned fiber laser, fiber pigtailed AOM, and the shut-off scheme. After the laser passed through the AOM, the light was mode-matched to the fundamental transverse electromagnetic (TEM_00_) mode of the cavity by lens 1, with a focal length of 50 cm. The ringdown cavity consisted of two mirrors separated by a distance of 39.4 cm, in which the input mirror was flat and the output one had a radius of curvature *R_c_* = 1 m. This gave a free spectral range (FSR) of 380.7 MHz. The reflectivity of each mirror was 99.94%, which gave a cavity finesse of around 5200. After the mode matching calculation, the beam radius on the flat input and concave output mirrors were 0.488 mm and 0.627 mm, respectively. The piezoelectric transducer (PZT) (HPSt 150/20-15/25, Piezomechanik GmbH, Munich, Germany) mounted on the output mirror was used to dither the cavity length to increase data points in a single spectrum for CRDS experiments. The transmitted light from the cavity was focused on the PD by using lens 2, with a focal length of 30mm. Lens 2 and the PD were tilted away from normal incidence to prevent the feedback coupling. A threshold circuit, working in a monostable design based on a 555 timer, was used to turned off the RF power to the AOM when the measured output voltage of the PD exceeded the preset value. The duration of the beam interruption, set to 0.5 ms, was controlled by the resistor and capacitor included in the threshold circuit. A 10-MHz, a 12-bit data acquisition card (PCI-6115, National Instruments, Austin, TX, United States) sampled the cavity transmission (i.e., ringdown signal), which was fitted to a single exponential decay by using the Levenberg–Marquardt method. The LabVIEW program was used to record data and show the absorption spectrum.

By careful alignment of the incident laser beam, the higher order transverse modes were reduced to less than 2% of the TEM_00_ mode. In this CW-CRDS experiment, a time delay of around 370 ns, including the response time of the threshold circuit, RF turn-off time, and the transit time of the acoustic wave traveling through the laser spot in the crystal, existed between the trigger signal to the AOM driver and the generation of the ringdown event. This delay results in amplitude variation of the ringdown event in the starting time, since the laser cutoff optical frequency is varied during this delay time, which does not seriously affect the intrinsic characteristics of the decay. According to Figure 1i, the measured shut-off time for the laser power was less than 6.15 ns, which can be neglected if compared to the ringdown time in the order of μs.

### 3.2. Observation of the Spatial Effect in the CRDS Measurement

In order to investigate the dependence of ringdown time on the spatial position of PD, a series of measurements were performed. The incident laser power to the cavity was about 2.5 mW, and the preset value of the threshold circuit was set to around 2 V for generation of ringdown events. During the experiments, the PD was assembled on a three-dimensional translation stage and moved from up to down in the cross-section perpendicular to the light propagation direction, with a fixed step of 50 μm. The movement of the PD always ensured that the detected light signal was higher than the preset voltage. In the horizontal direction, the PD was aligned at the center position by maximizing the detected light signal. In the following experiments, a 150 MHz bandwidth InGaAs PD (PDA10CF-EC, Thorlabs, Newton, NJ, United States) with the manufacturer-marked diameter of 0.5 mm was used.

**Figure 4:**
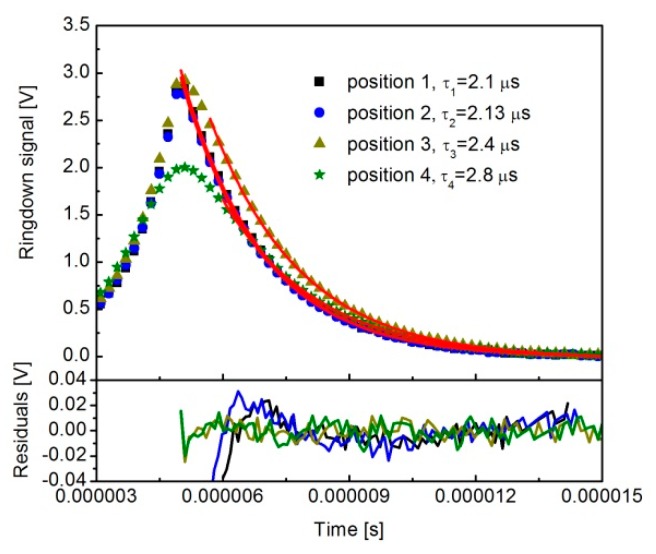
A group of gathered cavity decay signals when the laser focus of the PD was located in different positions. The red curve on the top of each decay signal shows the single exponential fitting and the residual is shown in the lower panel.

To confirm this spatial effect, we first evaluated the performance of the single exponential fitting model. Four individual ringdown decay signals gathered during the translation of the PD are shown in Figure 4, represented by solid squares, solid circles, solid triangle, and solid stars, together with the single exponential fits (red line) and the residuals (the lower panel). For clarity, the measured ringdown decays are displayed every at second point. Here, the laser beam at position 1 was tightly focused on the center of the photon-sensitive area of PD. The PD centers at positions 2, 3, and 4 were away from the laser focus in the vertical direction. However, the initial shut-off amplitudes for all the positions were larger than the preset threshold value of 2 V, due to the time delay between the trigger signal and laser shutdown, since the laser frequency was continued to scan during the delay. The initial shut-off amplitude of the decay at position 4 was smaller than other events, since the temporal response at this detection position is slower than those of others, according to the later analysis. The residuals indicate that the cavity decays were accurately demonstrated by the simple, single exponential fitting model.

**Figure 5:**
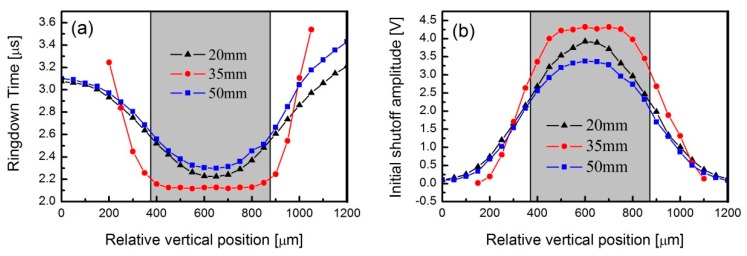
Measurements of (**a**) ringdown time and (**b**) initial shut-off amplitude as a function of relative vertical PD position when the PD was located behind lens 2 at a distance of 20 mm, 35 mm, and 50 mm.

Then, the distances between the PD and lens 2 were adjusted to 20 mm, 35 mm, and 50 mm, while the corresponding beam diameters at these distances were 490 μm, 57 μm, and 530 μm, respectively, which were measured with the previously mentioned beam profiler under the condition of laser frequency stabilization to the cavity mode via the Pound–Drever–Hall (PDH) technique. The measured ringdown times as a function of relative vertical positions are shown in Figure 5a. As can be seen, the measured ringdown times at all three distances varied with the translation of the PD, following the tendency of decreasing first and then increasing. It also shows that the farther the PD deviated from the laser beam, the larger the ringdown time was. Moreover, even if the laser injection position on the PD surface was identical, different ringdown times were observed at different distances from lens 2. Differing from the results at 20 mm and 50 mm, the ringdown time profile at 35 mm exhibited a steady platform (an average of 2.12 μs) in the range of about 400–800 μm, which was very close to the theoretical empty cavity ringdown time of 2.19 μs. In these measurements, the uncertainty of the ringdown time was in the order of tens of ns. Figure 5b presents the PD-detected initial shut-off amplitudes at those three distances. As with the ringdown time measurements at the distance of 35 mm, the amplitude profile also exhibited a constant value in a certain range. The dark shapes shown in two panels indicate the PD-sensitive area range.

### 3.3. Analysis of the Spatial Effect

**Figure 6:**
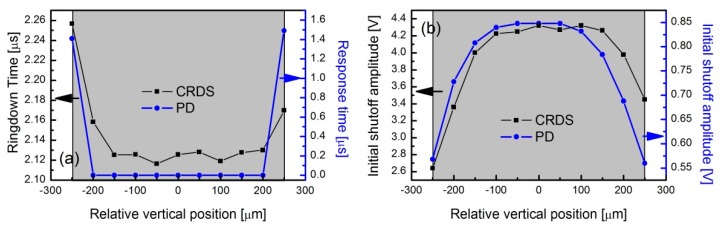
The measured response times (**a**) and initial shut-off amplitudes (**b**) as a function of relative vertical position for CRDS (solid squares) and PD (solid circles), respectively.

Figure 6 shows the measured ringdown time or response times in Figure 6a and initial shut-off amplitudes in Figure 6b for CRDS (solid square) and PD (solid circle), respectively. The data for CRDS is shown by the red solid circle in Figure 5. The data for CRDS show a small right shift in abscissa and a slightly wider platform range than those for PD because of the positioning error of the two measurements. The dark shapes shown in the two panels indicate the sensitive area range. It can be seen from Figure 6a that the measured response time of the PD and the ringdown time show similar behaviors over the relative vertical position (i.e., there is a range of relative positions for which both the PD response time and the ringdown time are at their minimums, and when the laser beam is moved out of this range, both responses get worse with further distance from the center of the detector area). Moreover, the ringdown time and the PD response time have almost similar dependences on the distance from the detector center, which gives indirect evidence that the variation of ringdown time (Figure 5) was caused by the PD response. Therefore, the ringdown time measurements are affected by both the temporal response of the PD and the true value of the photon lifetime. In addition, as can be seen from Figure 6b, the initial shut-off amplitudes are not only affected by the cavity, but also by the PD response. All of this shows that the measured decay time is the true cavity ringdown time when the light impinges upon the PD at a position at which the PD response is sufficiently fast. When the response functions of the cavity and the PD are on similar time scales, the resulted ringdown time measurements will be strongly influenced by PD response. All of this indicates that it is important to focus the light well on the central part of the depletion region of the p–n junction of the PD.

### 3.4. Influences on Trace Gas Detection

**Figure 7:**
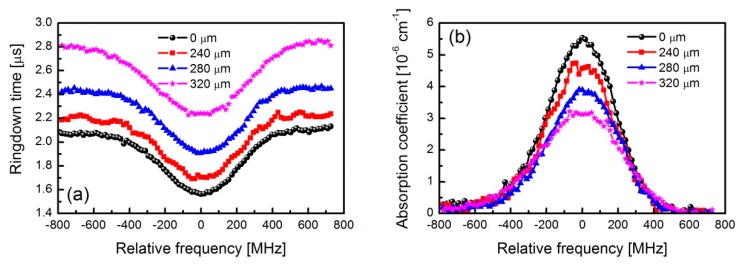
Measured absorption lines of C_2_H_2_ gas at the wavenumber of 6531.7805 cm^−1^. The cavity was filled with the 507 ppm C_2_H_2_ gas balanced with nitrogen and the total sample pressure was controlled to be about 1.88 mbar: (**a**) ringdown times; (**b**) absorption coefficients.

In order to evaluate the influences of this spatial effect on the spectral line shape measurement, the P(10)e rotational transition of the ν_1_+ν_3_ overtone band of C_2_H_2_ at the wavenumber of 6531.7805 cm^−1^ was analyzed [41]. In order to obtain a spectral signal, the laser frequency was tuned by a triangular wave at a frequency of 0.1 Hz, with an optical tuning range of 2 GHz. Meanwhile, in order to improve the spectral resolution, the cavity length was dithered at a frequency of 10 Hz, and an amplitude of cavity mode shift of around 400 MHz was applied to ensure a cavity mode in the dithering range. Another cavity with a length of 40 cm and a finesse of 300 was used to calibrate the spectral signal. The cavity was filled with the 507 ppm C_2_H_2_ gas balanced with nitrogen and the total sample pressure was controlled to be about 1.88 mbar. Figure 7a shows the measured absorption profiles expressed in ringdown times at different vertical positions when the detector was located at a distance of 35 mm from lens 2. In these measurements, the PDA10CF-EC detector from Thorlabs was used and the uncertainty of the individual ringdown time was in the order of tens of ns. The initial position (0 μm) was adjusted to ensure perfect alignment and coverage of the light spot. The distances of 240 μm, 280 μm, and 320 μm were implemented by moving the PD away from the initial position as was previously done in the translation. According to the beam focus size of 57 μm and the detector active area diameter of 500 μm, there should not have been any signal when the beam position was 257 μm away from the detector center. However, according to Figure 6b, the initial shut-off values still had a certain amplitude, which means the 1/e laser power outside the beam focus size played a role in this situation. As can be seen, the results at different positions have a similar profile and the profile depths at the line center are almost the same. However, the profiles have different offsets. By adopting the ringdown times of an empty cavity (i.e., 2.12 μs, 2.22 μs, 2.45 μs, and 2.84 μs, respectively) for the four different detected positions, the absorption coefficient α (in cm^−1^) can be calculated based on the formula $\alpha =\left(1/\tau -1/\tau_{0}\right)/c$, where c is the light speed, and τ and τ_0_ are the ringdown times with absorption inside the cavity and an empty cavity, respectively. The converted absorption profiles are shown in Figure 7b. As is shown, the peak absorption coefficients of the profiles are different, which is caused by the almost equal dip depth and different offsets in ringdown time profiles. Considering the Voigt broadening scheme, the calculated peak absorption coefficient of this transition is 5.47 × 10^−6^ cm^−1^, by adopting the line strength of 4.0 × 10^−21^ cm^−1^/(molecule⋅cm^−2^) from the high-resolution transmission molecular absorption (HITRAN) database, which has a stated uncertainty of 1%–2% [41]. The peak absorption coefficient of the profile at the position of 0 μm was 5.54 × 10^−6^ cm^−1^, which was very close to the theoretical calculation. The relative error of 1% was mainly induced by the inaccurate concentration of C_2_H_2_ gas, the imprecise control of gas pressure, and the relative uncertainty of the line strength. We can infer that the larger ringdown times obtained when the light spot did not impinge on the EDA of the PD was not the true value of the cavity decay rate. Consequently, the spatial effect will lead to inaccurate estimations of intracavity loss and gas absorption when the PD is not aligned perfectly.

## 4. Conclusions

Although multiple types of fast-response detectors are available for the measurement of transient signals, such as cavity ringdown decay signals, traditional PDs are also good options for the temporal time measurement due to their advantages of having a wide response bandwidth, being low cost, and easy to obtain. The inhomogeneous temporal and initial amplitude responses of different PDs were studied in this work, which caused a detector spatial effect in the CW-CRDS experiments. The spatial effect can strongly influence the spectral line shape and amplitude, which will introduce more errors into precise measurements of spectral parameters when using CRDS technique if the effect is not considered. Therefore, to obtain reliable and stable measurements in CW-CRDS experiments, the PD should be aligned perfectly, making sure that the laser beam is focused properly to match the size of the EDA of the PD. The CRDS experimentalists must pay attention to this effect, and one should check the response characteristic of the PD before CRDS experiments. The research into the spatial effects of ringdown event detection will help to precisely evaluate the reflectivity of super mirrors and trace gas concentrations, and will also serve as an alert for CRDS experimentalists.

## Figures and Tables

**Table 1:** Detailed information of three different photodetectors (PDs) employed in experiments.

Type of PD	Manufacturer	Bandwidth (BW)	Rising time (0.35/BW)	PD Diameter	Window
1611(DC output)	Newport	0.02 MHz	17.5 μs	0.1 mm	Ball lens (d = 1.5mm)
1811	Newport	125 MHz	2.8 ns	0.3 mm	flat
PDA10CF-EC	Thorlabs	150 MHz	2.3 ns	0.5 mm	flat
